# In Silico Gene Prioritization Highlights the Significance of Bone Morphogenetic Protein 4 (*BMP4*) Promoter Methylation across All Methylation Clusters in Colorectal Cancer

**DOI:** 10.3390/ijms241612692

**Published:** 2023-08-11

**Authors:** Daša Jevšinek Skok, Nina Hauptman

**Affiliations:** 1 Agricultural Institute of Slovenia, Hacquetova ulica 17, SI-1000 Ljubljana, Slovenia; dasa.jevsinekskok@kis.si; 2 Institute of Pathology, Faculty of Medicine, University of Ljubljana, Korytkova 2, SI-1000 Ljubljana, Slovenia

**Keywords:** DNA methylation, promoter region, bound region, colorectal cancer, CIMP

## Abstract

The cytosine–phosphate–guanine (CpG) island methylator phenotype (CIMP) represents one of the pathways involved in the development of colorectal cancer, characterized by genome-wide hypermethylation. To identify samples exhibiting hypermethylation, we used unsupervised hierarchical clustering on genome-wide methylation data. This clustering analysis revealed the presence of four distinct subtypes within the tumor samples, namely, CIMP-H, CIMP-L, cluster 3, and cluster 4. These subtypes demonstrated varying levels of methylation, categorized as high, intermediate, and very low. To gain further insights, we mapped significant probes from all clusters to Ensembl Regulatory build 89, with a specific focus on those located within promoter regions or bound regions. By intersecting the methylated promoter and bound regions across all methylation subtypes, we identified a total of 253 genes exhibiting aberrant methylation patterns in the promoter regions across all four subtypes of colorectal cancer. Among these genes, our comprehensive genome-wide analysis highlights bone morphogenic protein 4 (*BMP4*) as the most prominent candidate. This significant finding was derived through the utilization of various bioinformatics tools, emphasizing the potential role of *BMP4* in colorectal cancer development and progression.

## 1. Introduction

DNA methylation is a fundamental epigenetic mechanism that plays a crucial role in the regulation of gene expression [1]. It involves the binding of methyl groups by DNA methyltransferases to cytosine residues, resulting in the formation of 5-methylcytosine (5-mC) at cytosine–phosphate–guanine (CpG) dinucleotides [1,2]. In most somatic cells, the majority of CpG sites are methylated, with the exception of CpG islands located in promoter regions [1]. Notably, hypermethylation of these promoter regions often leads to the silencing of gene transcription [3]. Given that hypermethylation in promoter regions is an early event in colorectal cancer (CRC), it serves as a promising starting point for the identification of diagnostic methylation markers.

CRC is a prevalent malignancy worldwide and a significant contributor to global mortality rates [4]. The development of CRC involves a combination of genetic and epigenetic alterations in epithelial cells. Genetic abnormalities often include mutations in DNA mismatch repair genes and the APC regulator of WNT signaling pathway (*APC*) gene, which regulates the WNT signaling pathway [5,6,7]. In addition to genetic changes, CRC is characterized by widespread DNA promoter hypermethylation, leading to the silencing of tumor suppressor genes [5,6,7]. From a biological predisposition, CRC can be classified into two main subtypes: microsatellite instability (MSI) and microsatellite stable and chromosomally unstable (CIN), based on their genomic characteristics [8].

In 1999, the concept of the “CpG island methylator phenotype” (CIMP) was introduced, which characterizes the methylation of CpG islands in multiple genomic regions [9,10]. Currently, there is no consensus on the gene panel used to determine the CIMP status of a tumor. However, the most commonly utilized panel is the Wisenberger panel, consisting of the following genes: calcium voltage-gated channel subunit alpha1 G (*CACNA1G*), neurogenin 1 (*NEUROG1*), RUNX family transcription factor 3 (*RUNX3*), suppressor of cytokine signaling 1 (*SOCS1*), and insulin-like growth factor 2 (*IGF2*) [11].

CIMP tumors can be further classified into two subtypes: CIMP-H (CIMP high) and CIMP-L (CIMP low). CIMP-L tumors exhibit intermediate methylation levels and are often associated with mutations in the KRAS proto-oncogene, GTPase (*KRAS*) gene, with one to three genes from the aforementioned panel showing methylation [12]. On the other hand, CIMP-H tumors display high methylation levels, are significantly associated with mutations in the B-Raf proto-oncogene, serine/threonine kinase (*BRAF*) gene, and are predominantly located in the proximal colon. These tumors show methylation of more than three genes from the panel [11,12].

Recent studies have further expanded the classification of colorectal tumors based on their methylation subtypes. The Cancer Genome Atlas (TCGA) group identified four epigenetic subtypes: CIMP-H, CIMP-L, cluster 3, and cluster 4. The union of cluster 3 and cluster 4 is referred to as Non-CIMP [13]. Other studies, such as those conducted by Shen et al. [14] and Yagi et al. [10], identified three epigenetic subtypes and identified specific hypermethylated genes as markers. Additionally, Hinoue et al. identified four subtypes based on hierarchical clustering of DNA methylation, which exhibited high inter-tumor variability [15]. Among these subtypes, CIMP-H and CIMP-L were associated with *BRAF* and *KRAS* mutations, respectively. Tumors in the third cluster were associated with tumor protein p53 (*TP53*) mutations and were predominantly found in the distal colon, while the fourth cluster was enriched for rectal tumors with low rates of *KRAS* and *TP53* mutations. These subtyping approaches provide valuable insights into the heterogeneity of colorectal tumors based on their DNA methylation patterns and associated genetic alterations. Understanding these subtypes can have implications for prognosis, treatment selection, and the identification of potential therapeutic targets.

DNA methylation alterations have been found to primarily occur in the early stages of cancer, making them valuable early risk indicators for cancer development [16]. In the context of CRC screening, researchers have investigated aberrant methylation patterns in various genes within tissues and body fluids of CRC patients, identifying potential biomarkers for early detection. Notably, several genes associated with crucial signaling pathways have been studied as potential candidates. These pathways include the WNT signaling pathway, represented by *APC*, axin 2 (*AXIN2*), dickkopf WNT signaling pathway inhibitor 1 (*DKK1*), secreted frizzled related protein 1 (*SFRP1*), secreted frizzled related protein 2 (*SFRP2*), and Wnt family member 5A (*WNT5A*). Additionally, DNA repair processes have been investigated, with a focus on O-6-methylguanine-DNA methyltransferase (*MGMT*) and mutS homolog 2 (*MSH2*). Furthermore, cell cycle regulation has been a target of study, with cyclin-dependent kinase inhibitor 2A (*CDKN2A*) and cyclin-dependent kinase inhibitor 2B (*CDKN2B*) being of particular interest. Finally, the RAS signaling cascade has been explored, with emphasis on Ras association domain family member 1 isoform A and isoform B (*RASSF1A* and *RASSF1B*) as potential biomarkers in colorectal cancer research. These genes’ roles in these pathways make them promising candidates for understanding the mechanisms underlying colorectal cancer development and progression. Among these, promoter hypermethylation of cadherin 1 (*CDH1*) has been linked to CRC progression, suggesting its potential as a diagnostic tool for this malignancy [17].

Several DNA methylation biomarkers have shown promise for early CRC screening. Notably, N-Myc, downstream-regulated gene-4 (*NDRG4*), and bone morphogenetic protein-3 (*BMP3*) are tumor suppressor genes that can be utilized for early detection of CRC [18]. In different sample types, *NDRG4* methylation has been positively associated with CRC and adenoma, with sensitivity ranging from 27.8% to 81% and specificity from 78.1% to 91.7% [19,20]. *BMP3* promoter methylation analysis in blood, stool, and tissue samples has shown sensitivity ranging from 33.3% to 56.66% and specificity from 85% to 94% for diagnosing CRC and advanced adenoma [19,21]. Notably, three DNA methylation markers, including *NDRG4*, *BMP3*, and *SEPT9*, have been incorporated into FDA-approved tests for CRC screening [22].

In an attempt to identify a novel methylation marker by which to identify most CRC samples regardless of methylation cluster, we focused our analysis on DNA methylation data obtained from 332 colorectal cancer (CRC) tumor samples and 45 normal samples. Using unsupervised clustering, we identified distinct methylation subtypes within the CRC samples. Next, we found common methylation alterations across all four subtypes and assigned them to promoter regions, which belonged to 253 genes.

Among these genes, bone morphogenic protein 4 (*BMP4*) emerged as a particularly promising candidate based on further functional analysis. *BMP4* has been implicated in various biological processes, including cell differentiation, proliferation, and apoptosis. Its dysregulation has been associated with multiple diseases, including cancer. Our findings suggest that aberrant methylation of the *BMP4* gene promoter may play a significant role in CRC development and progression.

By identifying common methylation alterations in gene promoters across different CRC subtypes, our study provides valuable insights into the potential epigenetic markers and mechanisms involved in CRC. Further investigation of the functional implications of these aberrantly methylated genes, particularly *BMP4*, may contribute to a better understanding of CRC pathogenesis and potentially lead to the development of novel diagnostic and therapeutic strategies.

## 2. Results

### 2.1. Classification of CIMP Status

**Figure 1:**
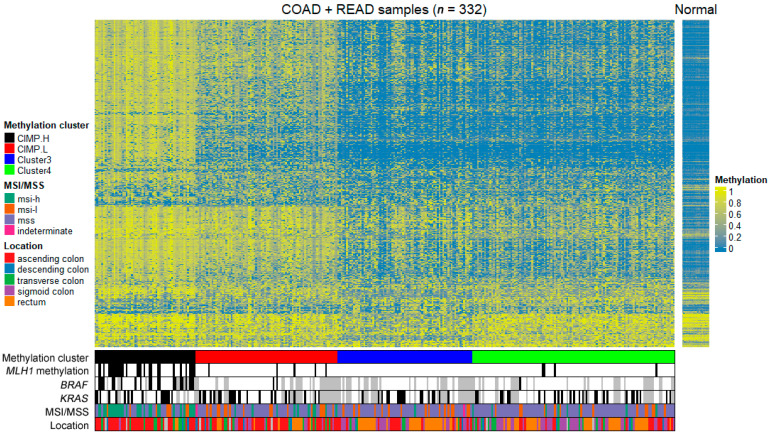
Heatmap of unsupervised hierarchical clustering of HM450 DNA methylation data of 332 tumor samples from colon adenocarcinoma (COAD) and rectum adenocarcinoma (READ) project in TCGA with some clinical and some genetic information. The color scale indicates the level of methylation: hypomethylation is blue and hypermethylation is yellow. CIMP.H, CpG island methylator phenotype—high; CIMP.L, CpG island methylator phenotype—low; msi-h, microsatellite instability—high; msi-l, microsatellite instability—low; mss, microsatellite stability; *MLH1*, mutL homolog 1; *BRAF*, B-Raf proto-oncogene, serine/threonine kinase; *KRAS*, KRAS proto-oncogene, GTPase; MSI, microsatellite instability; MSS, microsatellite stability.

To examine the differences in patterns of CpG methylation between tumor samples from different molecular subtypes, we performed hierarchical clustering of samples. We performed clustering based on the methylation data from HM27 using the recursively partitioned mixture model (RPMM) algorithm. Our goal was to identify subgroups within the dataset that closely matched the previously assigned clusters. To achieve this, we focused on 2757 probes that exhibited the highest variability in beta values among the tumor samples. By utilizing these probes, we obtained the best fit of clusters compared to the already assigned ones. This clustering model was then applied to HM450 methylation data to identify subgroups in the dataset. The hierarchical clustering dendrograms of HM450 methylation data (Figure 1) supported four distinct tumor groups: a heavily methylated cluster designated CIMP-H (*n* = 58), intermediate methylation levels cluster designated CIMP-L (*n* = 81), cluster 3 (*n* = 77), and cluster 4 (*n* = 116), with both clusters exhibiting low methylation levels. The clinical data from our clusters support previous studies wherein the CIMP-H subtype is enriched for *BRAF* mutation and *MLH1* methylation and is more commonly found in the ascending colon [23]. The cluster 3 and cluster 4 subtypes are more commonly found in the sigmoid colon and rectum.

The clinical and pathologic features of the patients and their tumors were summarized for further analysis. All 332 patients had information on age, gender, tumor location, MSI status, and methylation status on *MLH1* gene, and some also had mutational data available. For the purpose of classification, the mutational status of *BRAF* and *KRAS* mutations were used where available (Table 1). Consistent with previous studies, CIMP-H cluster is enriched in the ascending colon (38/58, 70%) and is associated with *BRAF* (V600E) mutation (21/49, 43%) and *MLH1* methylation (28/58, 48%) [16]. The sigmoid colon and rectum locations are commonly associated with cluster 3 (72%) and cluster 4 (65%).

The assignment of methylation cluster and all the clinical data used for each tumor sample and normal sample used in this study can be obtained from Supplementary Table S1. All further references to the CIMP status of our samples are based on our hierarchical clustering classification.

### 2.2. Promoters Methylated in All Clusters

After performing the cluster analysis, we compared the beta values of each cluster to the normal samples in order to identify probes with aberrant methylation. Due to the high density of the HM450k arrays, we were able to apply the Bonferroni correction method to assess methylation at all CpG sites within each cluster. To establish a higher confidence level for validating candidate methylation sites using experimental methods, we set two criteria for each site. First, the differential methylation threshold was set to 0.3; second, the Bonferroni-adjusted *p*-value was required to be less than 0.01. The final list of differentially methylated CpG sites were both statistically significant under the Bonferroni correction method and had a difference in the mean beta value greater than 0.3. The intersection of the significant CpG probes across all clusters yielded a list of 614 probes, and the results of this analysis can be found in Supplementary Table S2.

The probes significant among all clusters were subsequently mapped to 426 regulatory regions, including promoter and bound regions, associated with 253 genes. The observed variation in the number of regions and genes can be attributed to two factors. First, we considered both promoter-bound regions and predicted promoter regions, which can both be associated with the same gene. This overlap accounts for some redundancy in the gene count. Second, there are cases where multiple genes share common gene promoters, leading to a smaller number of unique genes despite a larger number of associated regions. These factors contribute to the observed differences in the number of regions and genes identified in our study.

**Figure 2:**
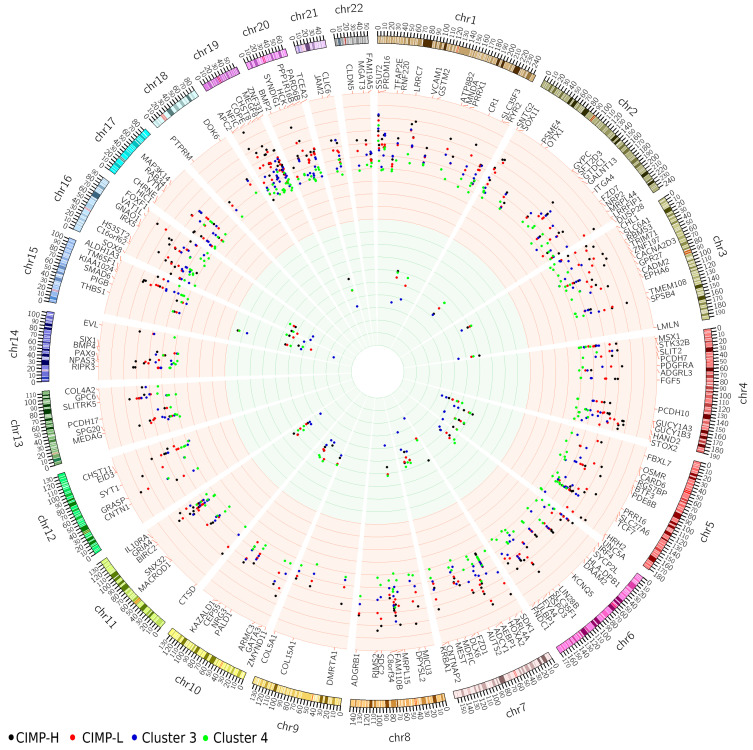
Visualization of averaged methylation level of promoter regions in all clusters, with corresponding gene names. Increased methylation is shown by the red background, while decreased methylation is represented by the green background. The clusters are visualized as colored dots, with the CIMP-H cluster displayed in black, CIMP-L in red, cluster 3 in blue, and cluster 4 in green. CIMP-H, CpG island methylator phenotype—high; CIMP-L, CpG island methylator pheno-type—low.

To derive a comprehensive view, the significant probes were extracted from the beta methylation value matrix and averaged based on their respective regulatory regions. In most cases, these values aligned with the criteria used in the differential methylation analysis, indicating a mean difference in beta values greater than 0.3. However, there were instances where this criterion was not met, primarily due to the presence of both hypo- and hypermethylated probes within the same regulatory region (Supplementary Table S3). These values have been illustrated in Figure 2, where the average methylation levels are depicted as increased (hyper) or decreased (hypo) compared to the average beta value in normal samples. Notably, we observed cluster-specific patterns of methylation events, with the CIMP-H cluster demonstrating the highest levels of hypermethylation.

The analysis revealed several gene promoters that had a high number of probes within the regions, indicating their potential significance in colorectal cancer. These genes include EYA transcriptional coactivator and phosphatase 4 (*EYA4*), alcohol dehydrogenase iron-containing 1 (*ADHFE1*), mitogen-activated protein kinase kinase kinase 14 (*MAP3K14*), and genes belonging to gene clusters: homeobox A cluster (*HOXA*) (*HOXA2/3/6*) and protocadherin gamma subfamily C (*PCDHGC*) (*PCDHGC4/5*). These genes have been extensively studied and proven to be hypermethylated and differentially expressed in various types of cancer [17,18,19]. Specifically, in colorectal cancer, *EYA4* and *ADHFE1* have already been validated as hypermethylated and down-regulated genes [24]. Furthermore, additional members of the same gene families have shown involvement in cancer. The *HOXA* cluster (*HOXA2/3/6*) has been identified as hypermethylated in breast and colon cancer [25]. *MAP3K14* has been found to be differentially expressed and hypermethylated in lung squamous cell carcinoma, where it regulates the NF-κB activity pathway and participates in NF-κB-inducing signaling through receptors of the tumor-necrosis/nerve-growth factor (TNF/NGF) family [26].

In total, there are 132 genes corresponding to 220 regulatory regions that have two or more probes mapped to them. Table 2 presents the top 15 regulatory regions with the highest number of significant probes mapped, which correspond to nine genes. Notably, all these regions exhibit hypermethylation compared to normal tissue methylation levels.

### 2.3. Functional Analysis

To explore the functional properties and relations between the genes involved in the colorectal cancer, we performed functional analysis with gene ontology (GO) annotations including biological process (BP), molecular function (MF), and cellular component (CC), as well as Kyoto Encyclopedia of Genes and Genomes (KEGG) pathways and protein–protein interactions.

The analysis showed that a total of 253 genes play significant role in 209 biological processes, 10 molecular functions, 4 cellular components, and 4 KEGG pathways (Table 3). The table presents the top-20 most significant biological processes, ranked by their strength scores. Among these processes, the one with the highest significance based on *p*-value is “Anatomical structure morphogenesis” (*p*-value 5.78 × 10^−11^). Conversely, when considering the strength score (1.51), “Ureter morphogenesis” emerges as the most significant biological process. Notably, a remarkable number of 200 genes are implicated in the process of “biological regulation”. The complete term list of gene ontology biological processes and all genes involved in each process can be found in Supplementary Table S4.

Regarding molecular functions, “Transcription regulator activity” exhibits the highest significance based on the *p*-value (0.0054). However, in terms of the strength score, “Cis-regulatory region sequence-specific DNA binding” is the molecular function that receives the highest score. In the cellular component category, the highest-ranking term is “Collagen-containing extracellular matrix” with a strength score of 0.49. The KEGG pathway analysis showed that 13 of the analyzed genes are involved in “Cell adhesion molecules (CAMs)” (*p*-value 2.87 × 10^−5^), and 6 in the “Basal cell carcinoma pathway” (*p*-value 0.0286).

### 2.4. Protein–Protein Interactions

**Figure 3:**
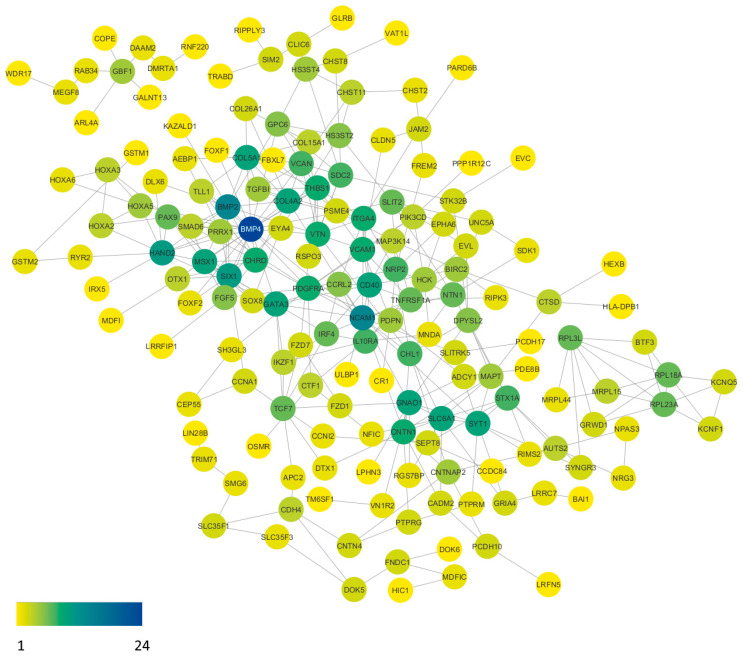
Protein–protein interaction network of aberrantly methylated genes in our gene list. The visualization was refined using specialized plugins within the Cytoscape software (version 3.10.0). Subsequent analysis of the network highlights *BMP4* as a standout candidate, engaging in 24 interactions with other proteins. Node coloration corresponds to interaction frequency, with yellow nodes denoting singular interactions, while the deep blue node signifies the network pinnacle, boasting 24 interactions.

To better understand the functional interactions between the identified genes with aberrant promoter methylation, the gene list was input into the STRING database to produce a functional association network based on their relationships, such as co-expression, co-occurrence, gene fusion, experiments, text-mining, and databases. A protein–protein interaction network (Figure 3) was constructed using our list of 253 genes. The network was further analyzed using the Cytoscape program plugins. Based on the network analysis of protein–protein interactions, it was found that *BMP4* emerges as the most promising candidate gene, highly connected with 24 interactions. The strength of their connectedness is presented in Table 4. Moreover, the table of all protein–protein connections and their combined score can be found in Supplementary Table S5.

### 2.5. Gene Prioretization Using Phenolyzer

**Figure 4:**
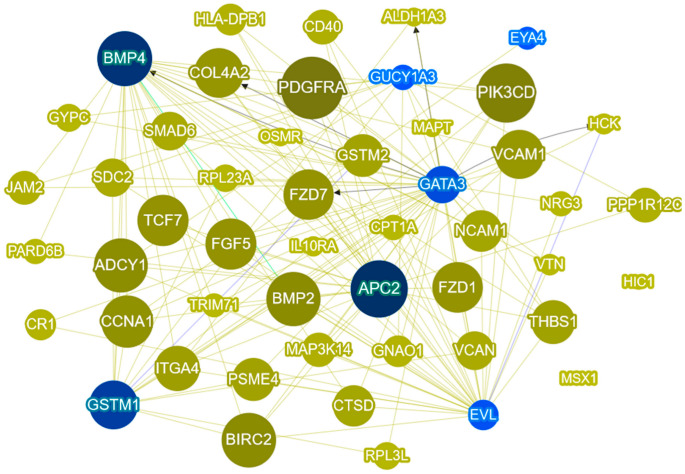
Phenolyzer network analysis of our 253 gene list and CRC terms. The most disease-relevant genes are shown as seed genes (blue) alongside the predicted genes. Yellow lines indicate that the two node genes are within the same biosystem; blue lines indicate that the two node genes have protein–protein interactions; and the black arrow line indicates transcription interaction.

Phenolyzer is an integrative tool which takes as input a discrete list of phenotype terms and generates a list of candidate genes weighted by their chance of being associated with the phenotype, even in the absence of any genotype data [27]. Phenolyzer uses three steps for data processing, described in the Section 3.5. Our list of 253 genes was used, each gene was given a score, and we generated a visual gene–gene network (Figure 4). The figure shows seed genes (blue), and predicted greens (yellow). The highest scores were given to platelet-derived growth factor receptor alpha (*PDGFRA*) (0.139), phosphatidylinositol-4,5-bisphosphate 3-kinase catalytic subunit delta (*PIK3CD*) (0.126), APC regulator of WNT signaling pathway 2 (*APC2*) (0.119), *BMP4* (0.112), baculoviral IAP repeat containing 2 (*BIRC2*) (0.112), and bone morphogenetic protein 2 (*BMP2*) (0.11). The genes with the highest scores are represented with the biggest round nodes.

## 3. Materials and Methods

### 3.1. Patients and Data

We utilized the TCGAbiolinks package [28] in the R programming environment to download both clinical information and DNA methylation microarray data included in the COAD (colon adenocarcinoma) and READ (rectum adenocarcinoma) project of TCGA. DNA methylation analysis was performed on level 3 data, which is already normalized and contains beta-value calculations, genomic coordinate, chromosome number, and Human Genome Organisation (HUGO) gene symbol for each CpG site on the array.

For our clustering model, we focused on methylation microarray data obtained from the Illumina Infinium HumanMethylation27k BeadChip (HM27). This dataset consisted of a total of 234 CRC samples, each containing DNA methylation data specifically generated from the HM27 array. Notably, these samples already had assigned methylation clusters, which were determined by the Cancer Genome Atlas Consortium [13]. Subsequently, we applied our clustering model to DNA methylation data obtained from the Illumina Infinium HumanMethylation450k BeadChip array (HM450). This dataset comprised a total of 332 CRC samples. Unlike the HM27 data, these samples did not have any assigned clusters from previous studies. Both microarray datasets were at data level 3, which included essential information such as beta-value calculations, HUGO gene symbols, chromosome numbers, and genomic coordinates for each targeted CpG site on the arrays. It is worth noting that the HM450 array targeted 482,421 CpG sites in the human genome. Additionally, we checked for the availability of mutation files for selected samples and downloaded them when accessible. For the purpose of differential methylation analysis, we also downloaded 45 normal samples that were included in COAD and READ dataset in TCGA.

### 3.2. Probes and Genes

The coordinates of protein-coding genes were downloaded from Ensembl, release 89 (http://www.ensembl.org/, accessed on 20 May 2023), while predicted promoter regions were obtained from the Ensembl Regulatory Build e89 [29]. The nomenclature of genes was unified according to the HUGO Gene Nomenclature Committee (HGNC) (http://www.genenames.org/, accessed on 20 May 2023).

We overlapped the HM450 probes mapped to the GRCh38/hg38 genome [30] with promoter regions and found their nearest genes. From the total of 482,421 probes, 93,794 probes are located within promoter region. The genes, where the transcription start position was within 5 kb of the mapped promoter region, were used for further analysis. *KRAS* and *BRAF* mutations were filtered from the mutation files. The mutations taken into account were in codon 12, 13 and 61 for *KRAS*, and p.V600E in *BRAF*. mutL homolog 1 (*MLH1*) DNA methylation status in each sample was asserted on the probe (cg00893636) located in the bidirectional *MLH1*/EPM2A interacting protein 1 (*EPM2AIP1*) promoter CpG island and closest to the current RefSeq *MLH1* transcription start sites. Beta value greater than 0.2 was taken as the threshold for methylation.

### 3.3. Unsupervised Clustering

For unsupervised clustering, we used the data from HM27 and HM450 arrays included in the COAD and READ projects of TCGA. The methylation data from HM27 has been previously clustered by the Cancer Genome Atlas Consortium, and the resulting clusters have been included in the samples’ information data [13]. In this study, we aimed to recreate the clusters of methylation data from HM27 and their clustering information. To achieve this, we utilized the recursively partitioned mixture model (RPMM) algorithm. Specifically, we focused on 2757 probes that exhibited the highest variability in methylation levels. By applying the RPMM algorithm to these probes, we obtained the best clustering results compared to already assigned clusters.

To cluster the methylation data from the HM450 array, we utilized a total of 332 CRC samples. Among these samples, 248 tumor samples belonged to the COAD project, while 84 tumor samples belonged to the READ project. The data from both projects were combined, and we performed unsupervised clustering using conditions determined based on the samples from the HM27 array.

The RPMM clustering was applied to both the HM27 and HM450 datasets. RPMM is a model-based unsupervised clustering approach specifically designed for DNA methylation measurements that follow a beta distribution ranging between 0 and 1. We preprocessed the data by removing probes mapped to the X and Y chromosomes, as well as probes containing “NA” values. Subsequently, we performed RPMM clustering on the 2757 probes that exhibited the highest variability in DNA methylation levels. The FANNY algorithm was used for initialization, which is a fuzzy clustering algorithm that assigns data points to clusters with varying degrees of membership, allowing for more flexible clustering. We used the level-weighted version of the Bayesian information criterion (BIC) as a split criterion for existing clusters during the RPMM clustering process. The BIC is a statistical criterion used to assess the goodness-of-fit of a model while penalizing complex models to avoid overfitting. In the context of RPMM, the BIC was used to determine the optimal number of clusters and to split existing clusters based on the level of DNA methylation. The RPMM clustering was implemented using the R-based RPMM package, which provides tools and functions to perform RPMM clustering on DNA methylation data. Overall, the RPMM clustering approach allows for the identification of distinct clusters of DNA methylation patterns within the datasets and explores the underlying structure of DNA methylation variability in the samples [31].

### 3.4. Statistical Analysis and Data Visualization

For differential methylation analysis, we used the same tumor samples as described above with an additional 38 normal samples from the COAD project and 7 normal samples from READ project. The analysis was performed in R (version 4.3.0) and Bioconductor software packages (version 3.18). For multiple comparisons, package TCGAbiolinks was used for the calculation of false-discovery rate (FDR)-adjusted *p*-values using Bonferroni correction [28]. Differentially methylated regions were calculated using data of each cluster compared to data of normal tissues. The threshold for difference in methylation between tumor and normal tissue was set to 0.3, and adjusted *p*-value threshold was set to 0.01. The Illumina Infinium DNA methylation β-values were represented in a heatmap constructed with R-based software package ComplexHeatmap (version 2.17.0) [32]. Averaged probes per promoter region were graphically represented with the Circos software (version 0.69-9) [33].

### 3.5. Functional Analysis

The STRING database (version 11.5) was used to identify gene ontology processes enriched within genes associated with differentially methylated CpG sites for 253 genes from the intersection of all four clusters [34]. The protein–protein interactions network (PPIN) were performed using the STRING database with Cytoscape plugin, which produces a functional association network using interaction sources such as textmining, experiments, database, co-expression, neighborhood, gene fusion, and co/occurrence.

The Phenolyzer tool (http://phenolyzer.wglab.org/, accessed on 8 June 2023) was used for the prioritization of our list of genes. The Phenolyzer processes the list of genes in three steps [27]. The first step is to search databases in the CTD’s (Comparative Toxicogenomics Database) disease vocabulary and disease ontology for a certain disease/phenotype term, interpret the term into multiple specific disease names, and find all the associated genes and related information in OMIM (Online Mendelian Inheritance in Man), Orphanet (a journal for rare disease), NCBI’s (National Center for Biotechnology Information) ClinVar, GeneReviews (an expert-authored, peer-reviewed disease descriptions), and GWAS (Genome Wide Association Studies) databases, then generate the seed gene set with conditional probability as scores. The second step is to grow the seed gene set in the HPRD (Human Protein Reference Database) protein interaction, NCBI’s Biosystem, HGNC (HUGO Gene Nomenclature Committee) gene family, and HTRI (Human Transcriptional Regulation Interactions) databases and retrieve an augmented gene set. The third step involves integrating all the information to score genes. The input can be one or several disease/phenotype terms, while users can optionally supply a gene list or genomic region to further trim down the candidate genes [27].

## 4. Discussion

In this study, we employed the high-density coverage of the HM450 methylation array to comprehensively investigate DNA methylation patterns in CRC. Leveraging this genome-wide approach, we identified four distinct methylation subtypes characterized by high, intermediate, and low levels of methylation. Our clustering approach was based on previous studies utilizing the HM27 array [13], and the clinical data associated with our clusters align with prior research findings. Specifically, the CIMP-H subtype showed enrichment for *BRAF* mutations, *MLH1* methylation, and a higher prevalence in the ascending colon [23]. In contrast, cluster 3 and cluster 4 subtypes were more commonly observed in the sigmoid colon and rectum.

The high density of the HM450 arrays enabled us to apply the Bonferroni test to evaluate methylation at all CpG sites of each cluster. While previous studies often set a low threshold (e.g., 0.1 or 0.2) for defining differentially methylated probes in genome-wide methylation array data, we set a threshold of 0.3 in our analysis. By intersecting the significant CpG probes and mapping them to promoter and bound regions, we obtained a list of 426 probes. These probes were then averaged within each promoter or bound region, increasing the confidence for validating candidate methylation sites through experimental methods. Specifically, we focused on 253 genes assigned to these regulatory regions. These genes were subsequently integrated into additional analyses to delve deeper into their interrelationship and their roles in CRC.

Among the gene promoters, several stood out due to having a higher number of probes within their regions. Notably, these included *EYA4*, *ADHFE1*, *MAP3K14*, as well as gene clusters such as *HOXA* (*HOXA2/3/6*) and *PCDHGC* (*PCDHGC4/5*). These genes have been previously studied and implicated in cancer, showing hypermethylation and differential expression [24,25,26].

To identify genes commonly methylated across all methylation subtypes, we employed two different approaches. Firstly, using protein–protein interaction analysis, we selected candidate genes based on the number of connected node genes. Among these candidates, *BMP4* emerged as a prominent gene. Secondly, we utilized Phenolyzer, a tool that prioritizes genes based on phenotype associations, and *BMP4* and *BMP2* were among the top-ranked genes.

Our comprehensive genome-wide analysis highlights *BMP4* as the most promising candidate gene with a potential role in the development and progression of colorectal cancer. *BMP4* is a member of the bone morphogenetic protein (BMP) family, which belongs to the transforming growth factor β (TGF-β) superfamily [35]. BMPs interact with type I and type II receptors, each possessing serine/threonine and tyrosine kinase activities. The BMP family can be further divided into subgroups based on structural similarities and their ability to bind specific type I receptors [36].

Intriguingly, BMP4, while primarily targeted toward the epithelial compartment, demonstrates a distinct localization within mesenchymal cells expressing α-smooth muscle actin [37]. A pivotal role of BMP4 is evident in its capacity to mitigate colonic inflammation and uphold intestinal homeostasis [37]. The perturbation of epithelial *Bmpr1a* has been shown to amplify BMP4 levels in the context of dextran sodium sulphate (DSS)-inuced colitis. Conversely, the inflammatory cytokines TNF-α and interleukin-1β (IL-1β) exert inhibitory effects on *BMP4* expression [37]. Recent investigations into DSS-induced colitis have unveiled dynamic expression patterns correlating with disease progression, notably observing an upregulation of BMP4 and Smad4 in the crypt during the early stages, followed by a downregulation in the later stages [38]. Counteracting the disease, exogenous administration of BMP4 recombinant protein emerges as a viable strategy, demonstrated to enhance epithelial proliferation by targeting an ID3 inhibitor and preserving Lgr5+ intestinal stem cells [38]. In addition, the transgenic overexpression of *BMP4* ligands along the intestinal crypt-villus axis yields contrasting outcomes—suppressing proliferation, hastening terminal differentiation, and impeding intestinal regeneration in the setting of DSS-induced colitis [39]. Furthermore, inhibiting BMP4 in intestinal stromal cells imparts a conducive environment for intestinal stem cell proliferation and the maintenance of intestinal equilibrium [40]. Evidently, a myriad of factors, including localization, concentration, and specific targets, intricately shape the duality of BMP4’s impact on the intestinal epithelium—proliferative or anti-proliferative [41].

Moreover, BMPs have been found to exhibit diverse roles in cancer progression. On one hand, BMPs have demonstrated inhibitory effects on the proliferation of gastric cancer, breast cancer, and prostate cancer cells [42,43]; on the other hand, BMPs have been reported to enhance the motility and invasiveness of various cancer cell types, including breast cancer, prostate cancer, and malignant melanoma cells [44]. In the context of colorectal cancer, the inhibition of *BMP4* has been shown to induce apoptosis in colorectal cancer cells by reducing mitogen-activated protein kinase (MAPK) activity in cell culture. Interestingly, increased *BMP4* expression has been discerned in the context of Colitis-associated colon cancer relative to nonneoplastic mucosa, underscoring its potential implication in disease progression [45]. Furthermore, an experimental BMP inhibitor, known as LDN-193189, has been found to suppress colorectal cancer formation in vivo [46].

Intriguingly, the intricate role of BMPs in colon cancer progression unveils a dualistic nature. Previous studies have illuminated a dichotomy, wherein *BMP4* overexpression has been correlated with the heightened invasiveness of colon cancer cells, as exemplified by [47]. Meanwhile, *BMP2* was shown to be hypermethylated in CRC [48] and has been observed to induce epithelial–mesenchymal transition, thereby potentiating the metastatic capabilities of colon cancer cells [49,50]. Furthermore, compelling evidence has emerged, as presented by Lorente-Trigos et al. [51], underscoring the BMP pathway’s pivotal role in nurturing the growth of primary colon tumors within an in vivo context.

Collectively, these findings coalesce to propose a nuanced conjecture regarding BMP’s role in colon carcinogenesis. The prevailing hypothesis posits a biphasic behavior of BMPs, wherein they potentially exert an initial tumor suppressor effect, subsequently transitioning to assume a tumor-promoting role. This nuanced perspective underscores the dynamic and context-dependent interplay of BMP signaling within the intricate landscape of colon cancer progression [52].

While previous studies have examined the impact of polymorphisms within BMPs or their expression on colorectal cancer (CRC) [53,54,55,56,57], only the *BMP3* gene has been previously identified as hypermethylated in CRC tissue [58,59]. In our comprehensive study of miRNA-target gene expressions in colorectal cancer, we have previously identified the overexpression of the *BMP4* gene in colorectal cancer tissue [60]. By utilizing the high-density HM450 methylation array and applying rigorous thresholds for differential methylation, our study provides valuable insights into the methylation patterns and potential biomarkers associated with CRC. The identification of specific gene promoters, including those previously implicated in cancer, expands our understanding of the epigenetic alterations underlying CRC development and may contribute to the development of novel diagnostic and therapeutic strategies. Our scientific contribution lies in adding an epigenetic dimension to the existing understanding of the role of *BMP4* polymorphisms and gene expression in relation to colorectal cancer. This aspect had not been previously substantiated or demonstrated in the scientific literature. These findings suggest that *BMP4* may also act as a tumor-promoting factor in the specific context of colorectal cancer.

The study is subject to certain limitations that should be acknowledged. First, the sample size, especially the relatively smaller number of normal samples, presents a limitation. A larger number of normal samples would have enhanced the statistical power and confidence in the results. Additionally, the availability of paired-tumor and matching normal samples with methylation data is limited, which restricts the ability to draw strong conclusions from such a small subset. Another potential limitation arises from the inherent biases introduced by the various bioinformatics tools utilized in the analysis. To address this concern, we adopted a strategy of employing multiple tools to cross-validate our findings. This approach helps us to minimize any potential biases and ensures the robustness of our results. Despite these limitations, the study offers valuable insights into DNA methylation patterns in colorectal cancer. By being transparent about the limitations, we hope to encourage further research and foster a more comprehensive understanding of the underlying mechanisms involved in this complex disease.

## 5. Conclusions

In conclusion, our study employed clustering analysis of the COAD and READ projects from TCGA using the HM450 array, enabling us to identify a set of genes exhibiting aberrant promoter methylation across all four methylation subtypes of colorectal cancer CRC. By utilizing various bioinformatics approaches, we have identified *BMP4* as the most compelling candidate gene. The comprehensive analysis presented in this study provides a valuable foundation for researchers investigating methylated promoter regions associated with colorectal cancer.

## Figures and Tables

**Table 1:** Clinical and genetic data of patients included in the study.

		Overall *n* = 332	CIMP-H *n* = 58	CIMP-L *n* = 81	Cluster 3 *n* = 77	Cluster 4 *n* = 116
Gender	Female	146 (44%)	25 (43%)	35 (43%)	36 (47%)	50 (43%)
	Male	186 (56%)	33 (57%)	46 (57%)	41 (53%)	66 (57%)
Age	Median	66	69	68	63	63
	Range	(31–90)	(33–88)	(37–90)	(37–90)	(31–90)
Subsite	Ascending	111 (34%)	38 (70%)	40 (51%)	15 (19%)	18 (16%)
	Transverse	41 (13%)	10 (19%)	11 (14%)	5 (6%)	15 (13%)
	Descending	13 (4%)	3 (6%)	4 (5%)	2 (3%)	4 (4%)
	Sigmoid	77 (24%)	1 (2%)	11 (14%)	23 (30%)	42 (36%)
	Rectum	80 (25%)	2 (4%)	12 (15%)	32 (42%)	34 (29%)
	No data	10	4	3	0	3
MSI status	MSI-H	48 (14%)	29 (50%)	10 (12%)	1 (1%)	8 (7%)
	MSI-L	54 (16%)	8 (14%)	16 (20%)	16 (21%)	14 (12%)
	Intermediate	2 (1%)	0	1 (1%)	1 (1%)	0
	MSS	228 (69%)	21 (36%)	54 (67%)	59 (77%)	94 (81%)
*BRAF*	Mutant	24 (10%)	21 (43%)	2 (4%)	0 (0%)	1 (1%)
	Wild-type	208 (90%)	28 (57%)	52 (96%)	46 (100%)	82 (99%)
	No data	100	9	27	31	33
*KRAS*	Mutant	83 (36%)	16 (33%)	23 (43%)	17 (37%)	27 (33%)
	Wild-type	149 (64%)	33 (67%)	31 (57%)	29 (63%)	56 (67%)
	No data	100	9	27	31	33
*MLH1*	Methylated	36 (11%)	28 (48%)	4 (5%)	0 (0%)	4 (3%)
	Unmethylated	296 (89%)	30 (52%)	77 (95%)	77 (100%)	112 (97%)

MSI, microsatellite instability; MSI-H, microsatellite instability—high; MSI-L, microsatellite instability—low; MSS, microsatellite stability; *BRAF*, B-Raf proto-oncogene, serine/threonine kinase; *KRAS*, KRAS proto-oncogene, GTPase; *MLH1*, mutL homolog 1.

**Table 2:** Genes with the highest number of significant probes in regulatory regions.

Gene Name	Mean Normal	Mean CIMP-H	Mean CIMP-L	Mean Cluster 3	Mean Cluster 4	Status	Probe Location	Number of Probes in Region
*EYA4*	0.09	0.62	0.59	0.59	0.48	Hypermethylated	promoter bound region	29
*EYA4*	0.09	0.62	0.599	0.60	0.49	Hypermethylated	predicted promoter region	25
*HOXA3*	0.33	0.73	0.72	0.74	0.69	Hypermethylated	promoter bound region	19
*PCDHGC5*	0.20	0.65	0.63	0.70	0.60	Hypermethylated	promoter bound region	16
*PCDHGC4*	0.20	0.65	0.63	0.70	0.60	Hypermethylated	promoter bound region	16
*HOXA2*	0.38	0.80	0.79	0.81	0.76	Hypermethylated	predicted promoter region	14
*HOXA3*	0.38	0.80	0.79	0.81	0.76	Hypermethylated	predicted promoter region	14
*HOXA6*	0.31	0.65	0.71	0.71	0.69	Hypermethylated	promoter bound region	12
*RIPK3*	0.10	0.67	0.52	0.51	0.45	Hypermethylated	promoter bound region	10
*PCDHGC5*	0.18	0.66	0.64	0.71	0.62	Hypermethylated	predicted promoter region	10
*PCDHGC4*	0.18	0.66	0.64	0.71	0.62	Hypermethylated	predicted promoter region	10
*RIPK3*	0.10	0.67	0.52	0.51	0.45	Hypermethylated	promoter bound region	10
*MAP3K14*	0.08	0.61	0.55	0.61	0.52	Hypermethylated	promoter bound region	9
*ADHFE1*	0.11	0.69	0.67	0.73	0.64	Hypermethylated	predicted promoter region	9
*ADHFE1*	0.11	0.69	0.67	0.73	0.64	Hypermethylated	promoter bound region	9

**Table 3:** Results of functional analysis for gene ontology and KEGG pathways (the biological process is represented via the first 20 processes).

Pathway ID	Pathway Description	Observed Gene Count	Background Gene Count	Strength	False Discovery Rate
**Gene ontology: Biological process**					
GO:0072197	Ureter morphogenesis	3	7	1.51	0.02
GO:0072189	Ureter development	5	17	1.35	0.0013
GO:0010463	Mesenchymal cell proliferation	4	16	1.28	0.0114
GO:0048557	Embryonic digestive tract morphogenesis	4	18	1.23	0.0152
GO:0042474	Middle ear morphogenesis	4	20	1.18	0.02
GO:0003177	Pulmonary valve development	4	21	1.16	0.022
GO:0048485	Sympathetic nervous system development	4	21	1.16	0.022
GO:0002053	Positive regulation of mesenchymal cell proliferation	4	25	1.08	0.0333
GO:0045992	Negative regulation of embryonic development	4	25	1.08	0.0333
GO:0061217	Regulation of mesonephros development	4	25	1.08	0.0333
GO:0003176	Aortic valve development	5	32	1.07	0.0113
GO:0060037	Pharyngeal system development	4	26	1.07	0.0364
GO:0010464	Regulation of mesenchymal cell proliferation	5	33	1.06	0.0124
GO:0048566	Embryonic digestive tract development	5	34	1.05	0.0137
GO:0042481	Regulation of odontogenesis	4	27	1.05	0.0385
GO:0048701	Embryonic cranial skeleton morphogenesis	7	48	1.04	0.0011
GO:0003180	Aortic valve morphogenesis	4	28	1.03	0.043
GO:0031128	Developmental induction	4	28	1.03	0.043
GO:0048704	Embryonic skeletal system morphogenesis	13	97	1.01	1.01 × 10^−6^
GO:0048483	Autonomic nervous system development	6	44	1.01	0.0057
**Gene ontology: Molecular function**					
GO:0000978	RNA polymerase II cis-regulatory region sequence-specific DNA binding	25	672	0.45	0.0041
GO:0000987	Cis-regulatory region sequence-specific DNA binding	26	701	0.45	0.0041
GO:0000977	RNA polymerase II transcription regulatory region sequence-specific DNA binding	30	878	0.41	0.0041
GO:0000981	DNA-binding transcription factor activity, RNA polymerase II-specific	32	1022	0.38	0.0041
GO:0000976	Transcription regulatory region sequence-specific DNA binding	32	1028	0.37	0.0041
GO:0003690	Double-stranded DNA binding	36	1156	0.37	0.0041
GO:1990837	Sequence-specific double-stranded DNA binding	33	1068	0.37	0.0041
GO:0003700	DNA-binding transcription factor activity	37	1238	0.36	0.0041
GO:0043565	Sequence-specific DNA binding	40	1331	0.36	0.0041
GO:0140110	Transcription regulator activity	43	1657	0.29	0.0054
GO:0000978	RNA polymerase II cis-regulatory region sequence-specific DNA binding	25	672	0.45	0.0041
GO:0000987	Cis-regulatory region sequence-specific DNA binding	26	701	0.45	0.0041
**Gene ontology: Cellular component**					
GO:0062023	Collagen-containing extracellular matrix	16	396	0.49	0.0453
GO:0098797	Plasma membrane protein complex	21	547	0.46	0.0196
GO:0031226	Intrinsic component of plasma membrane	44	1703	0.29	0.0196
GO:0030054	Cell junction	51	2075	0.27	0.0196
**KEGG pathways**					
hsa05144	Malaria	6	46	1	0.0095
hsa05217	Basal cell carcinoma	6	62	0.87	0.0286
hsa04514	Cell adhesion molecules	13	137	0.86	2.87 × 10^−5^
hsa04390	Hippo signaling pathway	9	153	0.65	0.0286

**Table 4:** Protein–protein interactions for *BMP4*.

Node 1	Node 2	Homology	Coexpression	Experimentally Determined Interaction	Database Annotated	Automated Textmining	Combined Score
*BMP4*	*CHRD*	0	0.062	0.723	0.9	0.988	0.999
*BMP4*	*BMP2*	0.961	0.076	0	0.9	0.901	0.907
*BMP4*	*MSX1*	0	0.062	0	0	0.854	0.857
*BMP4*	*SMAD6*	0	0.097	0.148	0	0.738	0.781
*BMP4*	*FGF5*	0	0	0	0	0.7	0.7
*BMP4*	*PAX9*	0	0	0	0	0.672	0.672
*BMP4*	*HAND2*	0	0.062	0.056	0	0.636	0.649
*BMP4*	*SIX1*	0	0.07	0.139	0	0.568	0.624
*BMP4*	*GATA3*	0	0.099	0.105	0	0.539	0.596
*BMP4*	*VCAN*	0	0	0	0	0.577	0.577
*BMP4*	*FOXF1*	0	0.07	0.056	0	0.551	0.571
*BMP4*	*TLL1*	0	0.064	0.243	0	0.406	0.542
*BMP4*	*GPC6*	0	0.096	0.153	0	0.441	0.535
*BMP4*	*THBS1*	0	0	0	0	0.532	0.532
*BMP4*	*PDGFRA*	0	0.062	0.07	0	0.48	0.507
*BMP4*	*NCAM1*	0	0.062	0.057	0	0.483	0.503
*BMP4*	*COL4A2*	0	0.088	0.246	0	0.304	0.479
*BMP4*	*OTX1*	0	0	0	0	0.455	0.455
*BMP4*	*COL5A1*	0	0.089	0.213	0	0.289	0.446
*BMP4*	*FOXF2*	0	0.075	0.056	0	0.407	0.437
*BMP4*	*VTN*	0	0.062	0	0	0.41	0.423
*BMP4*	*SDC2*	0	0	0	0	0.422	0.422
*BMP4*	*HOXA5*	0	0.053	0.057	0	0.403	0.42
*BMP4*	*SLIT2*	0	0.099	0.056	0	0.355	0.404

## Data Availability

All data used in this paper are available in the article and Supplementary Materials.

## Supplementary Materials

The following supporting information can be downloaded at: https://www.mdpi.com/article/10.3390/ijms241612692/s1.
